# Investigating the Perceptions of Care Coordinators on Using Behavior Theory-Based Mobile Health Technology With Medicaid Populations: A Grounded Theory Study

**DOI:** 10.2196/mhealth.5892

**Published:** 2017-03-21

**Authors:** Brittany Erika Sigler

**Affiliations:** ^1^ Department of Sociomedical Sciences Mailman School of Public Health Columbia University New York, NY United States; ^2^ Wellpass (formerly Sense Health) New York, NY United States

**Keywords:** communication, health behavior, Medicaid, mHealth, patient engagement, safety-net providers, text messaging

## Abstract

**Background:**

Medicaid populations are less engaged in their health care than the rest of the population, translating to worse health outcomes and increased health care costs. Since theory-based mobile health (mHealth) interventions have been shown to increase patient engagement, mobile phones may be an optimal strategy to reach this population. With increased development of theory-based mHealth technology, these interventions must now be evaluated with these medically underserved populations in a real-world setting.

**Objective:**

The aim of our study was to investigate care coordinators’ perceived value of using a health behavior theory-based mHealth platform with Medicaid clients. In particular, attention was paid to the perceived impact on patient engagement. This research was conducted using the patient-provider text messaging (short message service, SMS) platform, Sense Health (now Wellpass), which integrates the transtheoretical model (TTM), also called the stages of change model; social cognitive theory (SCT); supportive accountability; and motivational interviewing (MI).

**Methods:**

Interviews based in grounded theory methodology were conducted with 10 care managers to understand perceptions of the relationship between mHealth and patient engagement.

**Results:**

The interviews with care managers yielded a foundation for a grounded theory model, presenting themes that suggested 4 intertwined correlative relationships revolving around patient engagement: (1) A text messaging (short message service, SMS) platform supplements the client-care manager dynamic, which is grounded in high quality, reciprocal-communication to increase patient engagement; (2) Texting enhances the relationship between literacy and access to care for Medicaid patients, increasing low-literacy patients’ agency to access services; (3) Texting enhances communication, providing care managers with a new means to support their clients; and (4) Reminders augment client accountability, leading to both increased motivation and readiness to change behaviors, as well as an improved client-care manager relationship.

**Conclusions:**

Messaging platform features tied to health behavior theory appear to be effective in improving patient engagement. Two-way communication (supportive accountability), trusted relationships (supportive accountability, SCT), personalized messages (TTM), and patient input (TTM, SCT, MI) appeared as the most relevant components in achieving desired outcomes. Additionally, reminder messages were noted as especially useful in making Medicaid patients accountable and in turn engaging them in their health and health care. These findings convey suggested elements for inclusion in other mHealth interventions aiming to improve patient engagement in Medicaid populations.

## Introduction

### Untapped Potential of Medicaid Patients and Mobile Phones

Medicaid populations, that is those that qualify for the US government health insurance program for low-income individuals and families, are less engaged in their health care than the rest of the population, which translates to worse health outcomes and increased health care costs [1-2]. In this report, patient engagement will “denote a broader concept that includes activation; the (use of and participation in) interventions designed to increase activation; and patients’ resulting behavior, such as obtaining preventive care or engaging in regular physical exercise” [1]. Activation, in turn, is defined as “understanding one’s role in the care process and having the knowledge, skill, and confidence to manage one’s health and health care” [1]. This lower engagement is due to a complex number of structural and behavioral factors, including the lower access to care, and lower education levels and lower income levels, characteristic of Medicaid recipients [2]. Still, research indicates that Medicaid recipients use their cell phones as frequently as their non-Medicaid counterparts [3], suggesting that this may be an optimal strategy for reaching this medically underserved population. Since mobile health (mHealth) technology has shown potential to increase patient engagement in care delivery and chronic disease management across safety-net populations [4,5], cell phones may be able to be leveraged to reduce health disparities in this group.

### Collaboration With Minimal Evaluation

There is a paucity of data showing how mHealth interventions elicit patient engagement [6-9]. Whereas theory is being integrated in many digital health interventions after an initial disconnect between mHealth developers and researchers [10-14], there is a lack of real-world testing of these mHealth initiatives [15,16]. Determining how to best disseminate and implement evidence-based interventions among the intended audience (implementation science) can help “speed translation from discovery to application and public health benefits” [17]. Using data from the theory-based patient-provider texting (short message service, SMS) platform, Sense Health (Wellpass as of January 31, 2017) (Multimedia Appendix 1), there is a unique and timely opportunity to investigate how a theory-based mHealth intervention leads to engagement among a medically underserved population in a ‘real-world’ setting. The purpose of this research was to investigate whether and how integrating behavioral health theory into mHealth interventions leads to improved patient engagement in Medicaid populations, using a live texting platform.

### Communication in a High-Cost, High-Needs Population

The United States spent US $492.3 billion on Medicaid in the federal fiscal year of 2014 [18], which represents an annual growth of 5.2% since 2010 [19]. Patient engagement has become a crucial part of the discussion on how to reduce these costs [1], with the question becoming not whether to increase patient engagement, but rather to determine the most effective strategies to do so [20]. Low health literacy is one significant factor explaining the characteristic low engagement of Medicaid populations, which has implications for unequal access to care [21,22]. Health literacy is defined as “the degree to which an individual has the capacity to obtain, communicate, process, and understand basic health information and services to make appropriate health decisions” [23]. When health care providers ensure that communication is occurring at patients’ literacy level, they are able to increase their patients’ understanding of their own health [1,24], which may motivate patients to take a vested role in their own care [2,25]. In other words, patient-provider communication has the potential to trigger greater patient engagement. As a result, this increases access to care, which by definition, in addition to the ability to utilize care, entails: “Finding providers who meet the needs of individual patients and with whom patients can develop a relationship based on mutual communication and trust” [26].

The ubiquity of cell phones [3] and the rapid influx of mHealth initiatives into the public market [27] indicate that it is possible to make this type of communication a consistent part of the Medicaid population’s daily routine. Indeed, mHealth interventions have been propagated as the ultimate medium for the dissemination of behavioral health programs [4,9]. If research and practice in the mHealth field can be well integrated, this can facilitate important gains in public health impact. That is, mHealth technology might be able to mediate the thus far intractable relationship between health literacy and access to care, particularly in terms of care utilization and the development of meaningful patient-provider relationships among Medicaid populations by making patients more confident and capable managers of their own health.

### Using Sense Health to Fill a Research Void

Presently, the mHealth field is at a crossroads: the cell phone medium is at risk of losing its utility for public health delivery due to the dearth of research evaluating the effectiveness of the over 100,000 available, predominantly consumer-facing mobile apps [27,28]. Sense Health has integrated health behavior theory into the development of its mobile communication platform from the outset, thus presenting an opportunity to assess the implications of using a theory-based approach. The platform offers evidence-based coaching programs for chronic disease management and postdischarge compliance, appointment and medication reminders, and customized support, through 2-way mobile messaging. Specifically, the content of the platform is based on the transtheoretical model (TTM, also called the stages of change model) [29] and motivational interviewing (MI) [30], by tailoring semiautomated messages based on patients’ motivation and readiness to change. In practice, this means patients set their own goals [30] and messages are tweaked to encourage either cognitive exercises, such as simulation activities for less eager patients, or conscious action such as verbal commitments from the people who are keen to make positive changes to their behavior. The structure of the platform itself is also grounded in theory, including supportive accountability, which recognizes the importance of human support to amplify the effectiveness of mobile behavior change interventions [31]. To keep patients accountable to someone they trust, the platform connects them to their coach, that health expert who the patient already views as a steadfast source of support. This way, the platform acts to supplement existing communication. The 2-way conversation the coach has with the patient lets them jointly set the expectations of the program, which increases patient buy-in. In addition, by grounding the platform in trusting relationships and patient input, two constructs central to social cognitive theory, this texting platform inherently supports behavior change [29].

The findings on whether and how this theory-driven platform drives engagement can be used in the development and improvement of other mHealth interventions.

### Research Aims

The two primary goals in this research report were to determine the relationship between a theory-based texting platform and patient engagement through the perception of health care providers, specifically care managers, and whether these findings can be translated to other mHealth interventions. As high-need patients’ access to care is maximized with the assistance of care managers [21], their perspectives on using health technology are crucial to maximizing adoption of such interventions. This report aimed to:

(1) Examine the association between a theory-based texting platform and patient engagement

- Determine care managers’ perception of technology in the patient-provider relationship
- Determine care managers’ evaluation of patients’ opinions on technology use in a care-delivery setting
- Determine which technology components are related to patient engagement

(2) Make recommendations for how mHealth interventions can be refined to enhance engagement, and ultimately health outcomes, among Medicaid populations

(3) Outline recommended components of an evidence-based and theory-informed mHealth intervention

## Methods

### Overview of Research Design

**Figure 1:**
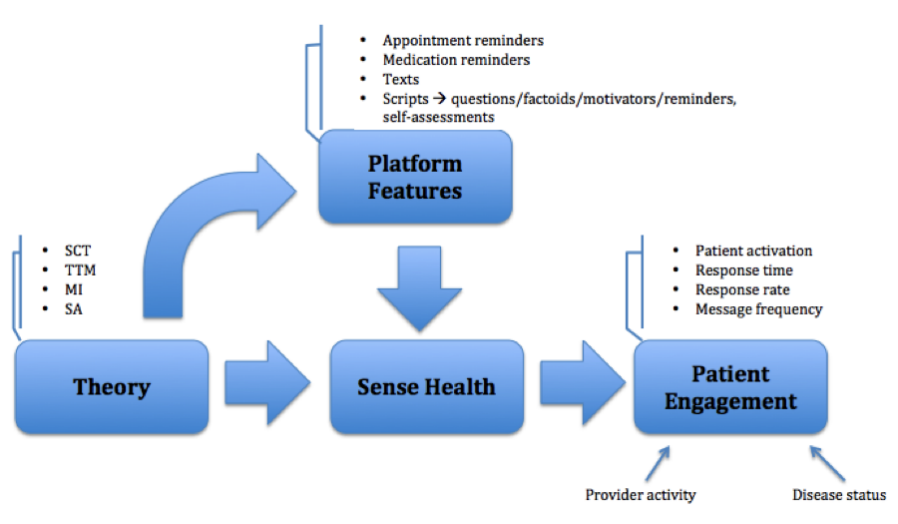
Conceptual model reflecting aims and study design.

This study presents qualitative research conducted through grounded theory-based interviews with care managers, which focused on their perceptions of integrating mobile technology with Medicaid clients. Grounded theory enables researchers to develop a theory to explain the phenomenon of interest [32]. Phrased differently, grounded theory is most appropriate for research that seeks to discover something new. The new theory is “grounded” in qualitative data garnered from those who actually experience the process: as the study progresses, the researcher’s initial exploratory question becomes refined until an understanding is reached regarding the topic of investigation [33]. That is, grounded theory involves “systematically discovering a theory from data” [34]. This is also when the information harnessed from interviews in the field will have reached the point of “thematic saturation” (also referred to as theoretical saturation) [32-34]. Saturation occurs when the information obtained becomes redundant, contributing nothing new or pertinent to the study’s findings [32,34]. Results can then explain existing practice and provide recommendations for future use surrounding the subject matter [32]. In this case, the phenomenon of interest is the perception of care managers on using mHealth technology with Medicaid patients. This approach is most appropriate for addressing this study’s aims, as mHealth stands to benefit from theories specific to its nuances, instead of trying to understand how mHealth is experienced through an amalgamation of existing theories created for less technological health intervention programs. As this phenomenon is not well documented, a grounded theory approach was used to structure the interview guides to help identify important factors and issues to guide future studies (Multimedia Appendix 2). The interviews focus on barriers to clients accessing care and care coordinators providing care. This would entail discussions of engagement; patient-provider communication; patient management of health, health literacy, patient motivation and readiness to change; integration of technology; and support and accountability of both the patient and provider. If this exercise indeed revealed that care coordinators perceived that aspects of technology improved patient engagement, additional inquiries on how they improve engagement would be added to the interviews. These relationships would be further probed in the context of the texting platform’s feature set to determine, as per the second aim, recommended components of an evidence-based and theory-informed mHealth intervention. The relationships that were explored in this study, as per the aforementioned aims and this study design, are visualized in a conceptual model (Figure 1).

Note that theoretical concepts and processes embedded in Sense Health were not discussed during user trainings, nor were they explained in the course of the interviews (Multimedia Appendix 2). The platform is typically framed during one-time trainings as a means to facilitate the user’s workflow. Prospective interviewees’ interactions with Sense Health employees are otherwise restricted to technical support. This study was approved under the IRB protocol IRB-AAAQ5254 by the Columbia University Medical Center Institutional Review Board.

### Recruitment

The data for this study came from interviews structured using a grounded theory approach with care managers who use the Sense Health platform. These care managers were recruited through email via a message crafted by the study author and sent by the provider experience manager at Sense Health. Recruited care managers were interviewed either in-person or over the phone, depending on their location and which method was chosen as most convenient for the interviewee. The approximately 30-60 minute interviews were audiorecorded either with a mobile phone or laptop computer depending on the setting of the interview. Respondents were required to provide verbal consent for study participation, including the recording. At the outset, participants were informed about the purpose of the study, the main research questions, and what information the author was planning to cover during the interview. Participants were then told they could skip a question or stop the interview at any time if they felt uncomfortable. After the recordings were professionally transcribed, the files were deleted.

All providers enrolled with Sense Health were eligible to be interviewed (Table 1). All providers were emailed the same day and the first 10 to respond were included in the qualitative component of the study. Respondents had three different job titles and were from six different organizations (Table 2). It was anticipated that 10 respondents would allow for theoretical saturation, and therefore to achieve the aims of this project, as dictated by Ward [33] who similarly used grounded theory to explore perceptions of a health care delivery setting with 13 interviewees, and Moola, Fusco, and Kirsh [35] who investigated perceptions of caregivers with a sample of 7. Guest, Bunce, and Johnson [36] systematically investigated the degree of data saturation over the course of thematic analysis in qualitative studies and found that saturation occurs within the first 12 interviews, with basic elements of themes present as early as the sixth interview, furthermore supporting the use of a small sample to sufficiently build a grounded theory. The same study purported that saturation may be the gold standard to determine sample sizes in qualitative studies. Time restrictions also influenced this aspect of the study design, as this research was originally conducted as one section of the author’s master’s thesis. If theoretical saturation were not achieved following the tenth interview, more would be conducted. Due to these time constraints, the author did not want to commit to an extraneous amount of interviews, beyond where theoretical saturation likely would be achieved, as per the aforementioned citations.

**Table 1:** Organizational characteristics of Sense Health users.

Organization type	Organizations	Individual users
Clinical trial^a^	4	5
Community-based organizations^b^	11	261
Homecare agency	1	8
Hospital	4	30
Nutritionist practice	1	2
Total	21	306

^a^ Clinical trial organizations are research studies using Sense Health.

^b^Community-based organizations encompass health homes, federally qualified health centers (FQHCs), and community mental health centers.

**Table 2:** Profile of respondents.

Job title (n)	Organization type (n)
Care coordinator (8)	Accountable care organization (1)
Patient health navigator (1)	Health home (3)
Care manager (1)	Community-based organization (2)

For context, all patients of the providers interviewed were Medicaid-eligible and enrolled from New York State. Hypothetical sociodemographic characteristics of the sample were drawn from Kaiser Family Foundation estimates based on the Census Bureau’s March 2015 current population survey (CPS: Annual social and economic supplements; Multimedia Appendix 3) [37]. New York Medicaid beneficiaries were predominately white or Hispanic, female, over 18 years, working full time, and up to 200% of the federal poverty level.

### Analysis

Dedoose version 7.0.23 (SocioCultural Research Consultants, LLC) was used to analyze the qualitative interviews. The author coded the provider interviews using grounded theory. For validation, the Sense Health provider experience manager reviewed the interview questions, coding methodology, and coding results, then provided feedback based on her daily interactions and familiarity with provider behavior. Analysis in grounded theory studies follows a rigid framework. As per the guiding literature on grounded theory, interview questions focus on understanding how individuals experience the process and identifying the steps in the process [32]. This emphasis facilitates axial coding later on, which revolves around identifying the core phenomenon, causal conditions, resultant strategies (ie, related actions and interactions), context, and consequences. Prior to axial coding, open coding takes place to allow for the formation of categories related to the phenomenon, here being care manager engagement with technology, by segmenting information. Properties of these categories are then identified. Development and refinement of these categories and the axial codes occurred in an external memo during the initial reading of the interview transcriptions (Multimedia Appendix 4). These codes were then inputted into Dedoose software to segment the actual interview transcriptions into these categories. This process is more tangibly illustrated in the Results section with interview excerpts. Next, selective coding entails connecting the categories, which also involves developing hypotheses that predict relationships related to the phenomenon, culminating in a “substantive-level theory” [32]. These connections are elucidated in the Results section of this paper.

## Results

### Grounded Theory Process and Interview Evolution

The Sense Health users who responded to the recruitment email varied in their activity levels on the platform, and also in their experience with the technology. However, their responses were surprisingly consistent. Sense Health was coded most frequently, as the interviewees zeroed in on texting through the platform without prompt, instead of discussing technology more macroscopically. This may be because Sense Health was the most tangible technology to the respondents and its role in engaging their Medicaid clients most visceral. None reported using other programs aside from electronic health records, which were purely used for documentation. As a result, these consistent patterns led to interview probes honing in on the Sense Health platform specifically, and engagement generally—independent of particular technology. The questions also evolved in an effort to better understand how engagement efforts were intricately tied to care coordinator bandwidth and the ability to integrate the Sense Health platform into the regular workflow. When regular patterns emerged concerning the role of care coordinators and their limited time to engage with their clients, that section of the interview guide was eliminated from proceeding interviews. The same was true for the questions concerning the typical demographics of the interviewee’s client population, their strengths and weakness in health care navigation, and the degree of communication. The final two sections of the interview guide lay the grounds for the majority of subject matter in the last 4 interviews: care coordinators’ perceptions of health technology, and views on their clients’ perception of technology. Indeed, only during the tenth interview, no new ideas were presented on these topics.

### Facilitating the Patient-Provider Dynamic

A very clear connection emerged between communication and engagement, whereby reliable, reciprocal communication between clients and care managers was indicative of the clients’ desire to engage in their health and with health care—whether the opportunity existed to do so at all. The importance of communication was much more prevalent than anticipated, which is largely due to the unexpected emphasis on the patient-care manager relationship:

It’s all about relationship-building. (...) You have to build a good relationship, and you have to build it from the get-go. That’s so important, because that contact, whether they respond well to you or not, that all depends on your relationship with them. (...) Because you’re working together with the client, you’re working together on their goals.

Care coordinator, Health Home

Texting acts to facilitate this contact, functioning as a supplement to regular communication and complementing the responsibilities of care managers. Whereas goal-setting and relationship-building will occur in-person or on the phone, text messages are an additional avenue of communication, and thus support (both for supporting the clients and for clients feeling supported), and are suggested as particularly useful for check-ins, reminders, follow-ups, and the exchange of small details:

Ya, like I’m like ‘oh, did you get the medical records from your doctor for the access-a-ride,’ that’s a transportation service, ‘do you need help with that?’—so we do have lengthy conversations via text, we can. But things like that, following up with stuff that they’re working on, or me filling them in, ‘I tried contacting that intake worker at that housing place, and they said you’re still on the waiting list.’ So a brief update like that I’ll text, it’s great.

Care coordinator, Health Home

In addition, care managers could save time by having multiple conversations at the same time through text. Text messages thus enable continuity of communication, making provider services more accessible:

One of the great things I do through Sense Health is I’m able to enter their questions rather than having them come in or call me. It’s easier for me to send them reminders, that for them to be like, ‘Hey, are you going to be there today?’ Or, ‘Hey, I have a quick question. I need this,’ or, ‘Can you remind me of the name of this or the number,’ so that I could just give it to them right away rather than to have them come in or have them call me. Especially because I’m always in and out of my desk, so if I see a message, then I can respond to them once I’m back.

Care coordinator, Health Home

Respondents emphasized that texting was a helpful means, but not the end goal. By maintaining a connection, the ease of communication also improved the relationship: care managers were able to remind their clients that they are there whether or not they have pressing needs. All respondents expressed that consistent communication and support were crucial to the care manager-client relationship and achieving care plan goals: “whether it’s jailhouse or chronic illnesses, medical illnesses, the biggest thing is support” (Care coordinator, accountable care organization [ACO]). Notably, whereas clients who are already engaged will take to using texting as a supplement to existing communication methods with their care manager, all interviewees pointed out that adopting texting allowed them to reach clients who were not partial to phone calls or in-person meetings:

...it’s definitely made getting in touch with some people easier. I think a lot of the care coordinators realized that over time. Where, at first, you kind of forgot that we had the service, so you’re still on the phone, you’re still calling and calling. Then, you know, two months go by and you hadn’t been able to reach someone, you’re like, ‘Wait. Why don’t I just text them?’ There you go, they respond.

Care coordinator, Health Home

But all care managers repeated throughout the interviews that all clients are different:

It all comes down to them, personally, again, are they willing to do something? Are they willing to make that change? The ones who will, you’ll see that change in them. Then the others, it’s been the same story for a year or two.

Care coordinator, Health Home

By establishing consistent reciprocal communication, a positive cycle of engagement would then be triggered for that patient—from managing their own care, to engaging with the health care system.

### A New Mode of Communication for Increased Support

Opening a new avenue for communication appeared to expand the boundaries of the existing provider-patient relationship, increasing the willingness of clients to engage. This connection was not predicated on replacing human interaction—which would be impossible in this high-need population—but instead on adding a new form of support grounded in existing trust:

They know that there is that trust because already they’ve given me, they’re allowing me to text them. It builds more trust and it builds more...Like, they’ll know that they can rely on me and so that’s a part of it that’s also good. They also, sometimes they like, ‘Oh, hey, thank you so much.’ It’s very receptive. Overall, it’s positive. I haven’t had any negative encounters.

Care coordinator, Health Home

The type of communication that occurs through texting is different, suggesting why adopting a texting protocol could newly engage patients. All care managers noted that clients will respond to, and engage with, different communication methods differently based on their varying needs. The dialogue was agreed to be less formal and more direct, opening a rapport with a more natural conversation tone and flow:

It’s maybe opened up more of a rapport with them. It seems like some people are more ready to share or to be texting these days than over the phone. You’ll get a smiley face. It’s just the texting generation we’re in today that people...You can get more of a feel of someone’s engagement sometimes through how they respond or just a little emotive. Yeah, I enjoy it. I don’t know if I’d rather...I mean I wouldn’t say (crosstalk) than speaking with them, but from an ongoing thing. If it’s just something simple, if I’m just trying to make a contact with them for the month to see how they’re doing, yeah, I’d rather use Sense Health, but if I really do have to speak with them or schedule something, obviously the phone platform would be great. Maybe a follow-up with them through Sense Health. Like, hey, what’s going on for tomorrow?

Patient health navigator, Health Home

With control over the pace and content, clients could also communicate at their own speed, mitigating literacy barriers that would occur through other means (particularly in in-person interactions with health care providers). One care coordinator from a health home explained issues associated with health literacy and the resultant need for quick communication between them and their client at 2 different points in their interview:

They’re literally sitting in the waiting area for hours, they’re hungry, it’s for a follow-up they don’t understand...

...some clients, they won’t communicate another symptom they’re having that they felt comfortable telling me. Maybe they don’t like the side effects of one of their psych meds and they won’t talk to their doctor about it.

Clients were perceived, as a result, to be more open, share more details, and contact their provider more often through texts, leading to increased engagement.

Most care coordinators were selective in introducing the platform to those who they thought would most benefit. These clients were either perceived to be more adept at texting, they were not receptive to phone calls, and younger. As per a patient health navigator at a health home:

The younger generation, and by younger, I’m going to say like 40 and under. They’re more inclined to wanting to use it, or more open-minded to using it.

All care coordinators shared that among the clients presented with the option to communicate through texts, acceptance was nearly unanimous:

I think it’s (texting) just so commonplace now that they just accepted. ‘Oh, okay.’ (...) If they do decline it’s just because some of them don’t even have a cell. The older people, some of them don’t even have a cell. (...) I don’t think I’ve ever really had the pushback of no I don’t want that because it’s they’re meeting with us and they’ve agreed to this program this is free as long as they’re cooperating and using it. Most of them have at least the intention of wanting to do something differently.

Care coordinator, ACO

Nine and a half times out of ten, they say, ‘Yeah, that's great. Yeah, I can text. That's fine.’

Patient health navigator, Health Home

As with the care coordinator quoted above, those who did not have positive perceptions of texting were often older clients with vision issues and lack of technology skills, or those with poor literacy (often due to language barriers), and security concerns. From the provider perspective, they similarly experience more control over text communication, with the liberty to respond to clients when they are able—both in terms of availability of time and requested information:

When they’re trying to get a hold of you, or if they just have something quick to say or ask, it can just be easier because I can see that. I can get that message while I’m on the road. I can respond when I stop somewhere. Instead of having to call back to get a full explanation of what their needs are, what the issue could be, I can just read it right then and there. Then actually start working on that, and then get back to them with an answer rather than call back to find out. Then I go and work on it. Then I call back and tell them what I’ve done.

Patient health navigator, Health Home

This newfound control facilitates the regular workflow, and as a bonus prevents unwanted drop-in appointments. The ease of use and utility for engaging patients prompted care managers to express that they wished more clients would use the texting platform, despite the time-consuming consent process. Other nominal concerns regarding the platform included the length of texts (a character limit could impede the clarity of messages), and hesitancy with clients’ plan (do not want to take up limited texts available) and phone (phones with T9 keyboards are more difficult to text with).

The ubiquity of cell phones was routinely acknowledged, as well as the need to embrace that reality and work it to the providers’ and clients’ advantage through “positive,” productive use of a familiar tool. As shared by one care manager:

Since we are in a world of technology, the majority of them with the exception of a couple clients of mine who are quite a bit older, they all prefer. This is the day and age where technology is what we know.

Care coordinator, ACO

Medicaid clients were also noted for their constant mobility, with a lack of consistent housing and employment. In the words of a health home care coordinator, communicating through text messages is a beneficial “organizational tool” for these clients given these circumstances: “text messages with medication and appointment reminders act as a pseudo-calendar.” Moreover, the conversation itself acts as documentation that could be used as a reference point by both parties, and which can visually demonstrate client progress through their messages. Almost all interviewees believed that this functionality increased patient accountability, “especially given the ability to automatically follow up” (Care coordinator, Health Home). Despite this advantage, benefits could not be actualized until social and financial needs, especially housing needs, were addressed: “if you’re homeless, that’s going to affect your health. I know your priority is to get a roof over your head” (Care coordinator, Health Home). Only at that point, providers explained, would clients become engaged in their health and interested in interacting with the health care system. Furthermore, they elaborated that clients would be less interested in the “whys” of their health, and more in the “hows”: as in, they would be more concerned about how to deal with their illness, rather than why they have it.

### Increasing Patient Accountability and Positive Behavior Change Through Reminders

If clients are willing to change their behavior, then reminders can increase engagement with their health and the health care system, and health management, especially in terms of medication adherence. This is because all respondents perceived an increase in client accountability and motivation after assigning appointment and medication reminders:

It does give them a little bit more of, like, ‘Okay, I have to do this because she’s going to know if I don’t.

Care coordinator, Health Home

...because if they tell you they have an appointment coming up and you remind them to go, they’re on top of things, so they’re able to manage their health better. Again, that’s something that I would only know if they inform me.

Care coordinator, Health Home

Care managers attributed these desirable outcomes to their ability to tailor the frequency of the reminders based on individual client needs, preferences, and readiness to change. With the ability to automate allowing for perfect consistency, the reminder features allow care managers to move the burden of reminding, and following up with, clients about appointments to the platform:

(One) client is very forgetful about his appointments and he’s like, ‘I need you to set the reminders.’ Then he follows up, it’s like, “Okay, I’m going to go.’ Or he follows up with me, ‘This is what’s done.’ It’s really good because I can just send a text message, they’ll send it automatically. I don’t even have to do it. Then they’ll know, ‘Okay, I have to take care of this.’

Care coordinator, Health Home

Texting was found to trigger positive health behavior change, increasing patients’ management of their health condition by increasing appointment attendance via reminders. Specifically, the reminders were the catalyst acting to increase clients’ ability and readiness to change their behavior. Once at their appointments, clients are prompted to have follow-up conversations and questions with their care manager—and learn things they would not have taken the time to understand, or recognize, otherwise:

...a lot of them (...) went from not going to any appointments (for) their health, their mental health, everything was pretty bad, to going to (these) appointments consistently and having some of the problems that they were having prior help or fixed. Or at least decreased. It shows them the benefit of going to their appointments. The appointments aren’t made just because. I’ve seen it where the medications weren’t working. If they didn’t go to the appointment, then they wouldn’t be able to have their medication switched. Whether increased or decreased or just changed the medication altogether without that being there. It definitely helps a lot.

Care coordinator, ACO

Indeed, there was a positive feedback loop between access to services and engagement with both health and providers, whereby going to the doctor encouraged further engagement, and engagement in health and with a care manager encouraged clients to access services. Similar trends were apparent with medication compliance. When clients would respond that they took their medication after receiving their reminder, it would increase their motivation to keep making positive changes, as they would see the positive effects:

I have a client who I, basically, set up reminders all the time to take his medication. He texts me when he’s done taking the medication. He answers, ‘This is great, I love that I actually can let you know. I know that I have to take it, and then sometimes I oversleep or forget, and it’ll send me another message just to make sure, just to remind because I haven’t text ‘done’. It means that I haven’t taken the medication.

Care coordinator, Health Home

Sometimes being prompted, ‘now is the time to take your meds,’ that helps the people that really want to try to make themselves better.

Care coordinator, ACO

The ones who I have their medication reminders for, like seeing that change in themselves, they’re happy about. I guess it kind of helps them just be happier about what’s going on in their life.

Care coordinator, Health Home

Indeed, all care managers perceived motivation as the key to patient engagement. The “yes” response also would reinforce trust in the care manager-client relationship: the 2-way communication acts to keep both the care manager and client accountable, expanding the accessibility of the care manager, and ensuring that clients are doing what they are asked or supposed to. Whereas there was a split among providers on who is made more accountable, all say there is an effect, whether one party, the other, or both.

Perceptions were inconsistent on the value of tailoring responses. Some believed that personalizing messages was helpful in engaging patients, enhancing customization, triggering client memory, and proving that the messages were not automated:

It makes a difference, it does. Some of them will actually ask you, ‘Is this really you Ashley? Are you talking to me or is this automated?’ Or they’ll call and ask and I’ll say no I’m standing right in front of the computer and I’m the one that’s texting you.

Care coordinator, community-based organization

Others believed that the content was helpful regardless of how it was presented:

I think most of them are happy that they get a reminder or they get a text. It’s not so much of what the context of it is that the fact they were reminded or...A lot of them think that it comes from us personally, so the fact that they were reminded, having taken the time and trouble to remind them of something, is what helps us support them.

Care manager, Health Home

Interestingly, all respondents seemed to believe that it was more important for a text to come from someone trusted, than that the text is personalized, given that a supportive relationship yields improved engagement.

### Emergent Grounded Theory Model

**Figure 2:**
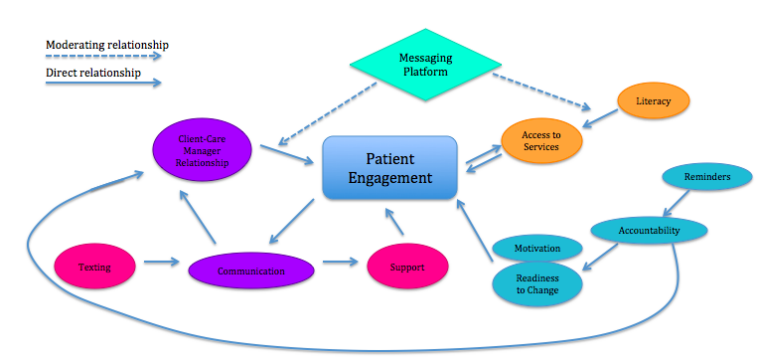
Grounded theory model resulting from interviews.

Foundation for a theory to explain patient engagement emerges from axial coding, suggesting correlative relationships among four different components of the patient-provider experience. This is illustrated in the model using different colored ovals (Figure 2). These relationships were based on themes that arose in the interviews. Using a texting platform acts to amplify two existing relationships, whereas introducing a new means to provide support and encourage accountability. The most prevalent component seems to be that technology can strengthen the relationship between the client-care manager dynamic and engagement, by facilitating the provider’s ability to engage their patients, and that this relationship is founded on the quality, quantity, and reciprocity of communication. Integrating a messaging platform into the care coordinator workflow also interferes with the relationship between literacy and access to care among Medicaid patients, whereby texting, as a feature, gives patients with low literacy increased agency to access services. Finally, the messaging platform introduces new tools to care managers, providing them with both a new means to support their clients, making communication more accessible, and a tool, namely the ability to automate reminders, to motivate clients by increasing their accountability to their health.

Care managers were consistent in explaining that their client response rates to contact attempts varied based on need for assistance. Specifically, they said that most clients require several contact requests before completing a touchpoint. The interviewed care managers were explicit that, beyond maintaining engagement in previously engaged clients, implementing texting through a robust platform also triggered increased engagement in previously unmotivated clients. TTM could help to explain this new engagement, given that everyone responds to different methods for changing their health behaviors.

There was indication that a texting platform may improve health literacy in care managers’ clients, leading to better access to care. Inherent in their job description, care managers mediate poor health literacy in clients by helping clients navigate their health and health care, and texting facilitates their ability to do so. The platform helps care coordinators’ clients better understand how to manage their health (in terms of medication adherence), increasing client knowledge, and making clients more accountable to their own health and more independent in their interactions with the health care system—all aspects of high health literacy. This encourages clients to utilize care and to develop fruitful relationships with their care providers.

Providers mentioned minor differences in ease of use of the texting platform based on client background. Whereas demographic information particular to Sense Health users was not available, as it is not collected, care managers were unanimous in their interviews that they found no difference in engagement based on race, gender, or ethnicity. They did, however, note that older clients (defined differently by different respondents, ranging from 50+ to 70+ years), and those inexperienced with texting, had the most difficulty engaging in their health through text messages.

Clients would respond favorably to motivational messages but care managers perceived reminders to have greater utility in engaging their patients. The care managers shared that reminder messages were the most effective feature of the texting platform for engaging their patients. That is, appointment and medication reminders would encourage compliance, triggering a cycle of positive behavior, interest in self-care, and follow-up conversations requesting more information. In other words, reminders would act as a trigger moving clients forward to the next “stage of change,” as per TTM. Providers perceived that texts amplify their ability to support their patients, whereas reminders make clients more accountable. This suggests that integrating the theory of supportive accountability was useful in attaining desirable platform outcomes. These outcomes were only possible, though, due to previously established trusted relationships between the care managers and their clients, and their focus on goals crafted with the patient—highlighting the effectiveness of two constructs of SCT.

Scripts were perceived as being ideal for improving knowledge of clients’ health conditions, although no respondents had used that feature at the time of the interview. As the interviewed care coordinators did not use this feature, further research is needed to support the integration of motivational interviewing, which is tied to the goal-setting aspects of the platform. This inclination, though, aligns with the benefit of integrating motivational interviewing and TTM, as scripts incorporate these theories’ constructs more than other features. Some care managers also expressed benefits to personalizing texts, and tweaking reminder messages’ content and repetition frequency based on client needs, as suggested by TTM, in order to maximize engagement.

## Discussion

### Recommendations for Practice and Research

The results of this paper suggest several factors for consideration when designing mHealth interventions to enhance engagement and ultimately health outcomes in Medicaid patients. These suggestions, however, are based on a small sample and for a specific use case and thus should be interpreted accordingly. Evidence-based, theory-informed mHealth initiatives may consider incorporating 2-way communication to maximize patient accountability to their health management, as suggested by the theory of supportive accountability, and supported by the aforementioned findings. Similarly, dynamic mHealth interventions that aim to change health behavior could further investigate the benefit of using trusted health coaches to cement a relationship between the “provider” and “patient” before working on health goals. This component is tied to SCT, as well as supportive accountability, and was also emphasized in this study’s results. Finally, the ability for even minor customization to health message content based on individual patient needs, preferences, and readiness to change, recommended by TTM, and the facilitation of patient agency, recommended by SCT, were demonstrated to be beneficial and are components encouraged for future interventions.

Future research can further develop the model that has emerged from this study, and help better understand the use of technology in this particular environment. The next steps for this research project, ideally, would be to interview care managers at each provider practice or health system that implements Sense Health, or any text-based technology. It would also be useful to conduct focus groups within one specific location, and with participants from various locations, to facilitate a comparison of viewpoints whereas also eliciting a more substantiated perspective of overarching themes. Provider discussions could also include a component focusing on the return on investment of implementing mHealth interventions. Finally, a much larger project would also involve patients, most likely with questionnaires since they are a vulnerable population, investigating their perspectives on using technology to manage their care, to elucidate more than just the care managers’ perception of client perspectives. This study could also be scaled up even further across numerous theory-informed mHealth interventions which would yield more representative findings of what components and which theories are most effective in improving patient engagement outcomes.

### Limitations

The validity of the interpretation of the findings presented here is subject to multiple threats. The interviews regarding patient engagement were from the care managers’ perspective, instead of the patients themselves, which by nature makes those findings subjective. Moreover, given that interviews were conducted with the first 10 respondents to the recruitment email, their participation might be biased. They might have volunteered to be interviewed due to unusually strong opinions concerning the platform, a desire to cultivate a positive relationship with Sense Health, or an inclination to verbalize their experiences with their patients on the platform. Such volunteer bias could have influenced the results through responses that were either more positive or more negative than average. With the possibility for the respondents’ user experiences deviating from the average in both directions, overall findings were likely representative of the general provider population enrolled in the texting platform.

There was also risk of researcher bias: as a former company intern, the author had expectations of how the care managers would respond according to their knowledge of user activity data on the platform. Given that Sense Health recruited the care managers interviewed, this gave the care managers certain assumptions about the purpose of the study. The author had never interacted with the interviewees prior to this research, and was not privy to their personal usage of the platform. Interviewees were ensured that their responses would remain anonymous and that their answers could not be traced back to them. The purpose of the study and the interviews was reiterated in both the recruitment email and at the outset of the interview sessions.

### Comparisons With Prior Work

The results of this study are consistent with others [1,9,12,38-42], which suggest that incorporating theory will maximize the effectiveness of mHealth behavior change interventions. The results also support findings [5,43] that cell phones can be leveraged to improve patient engagement in Medicaid populations through patient-centered care models [1,24], improved health [25], and management of chronic disease [4,44,45]. Finally, the findings presented here also fulfill the call to action to the scientific community to conduct implementation studies examining the effectiveness of theory-informed mHealth interventions in real-life, uncontrolled settings [5,9,11,15,16,39,42,46,47], as well as identifying recommended mHealth intervention characteristics [8].

### Conclusions

Overall, the findings support the intended aims, helping build an assertion where integrating health behavior theory in this mHealth intervention increases patient engagement in the interviewed care managers’ Medicaid clients. This adds to the existing body of literature on integrating health behavior in digital interventions, using mHealth to engage patients, and on methods of engaging patients in Medicaid populations. By addressing numerous topics in mHealth and health behavior research simultaneously, this study supports further development of theory-based mHealth interventions targeting patient engagement, while also substantiating their adoption and use in Medicaid populations. The limitations of this study also provide guidance for future studies and mHealth initiatives, helping to avoid the flaws revealed in this project’s methodology and results. This is significant as this area of implementation research is in need of greater attention. Without initial forays in behavior-theory guided mHealth implementation studies, there will be no foundation for increasingly rigorous efforts. With further rigorous implementation research, eventually systematic reviews may also be conducted, which would greatly benefit the field. The implications of this projected trajectory are important: with increasing evidence for the benefit of incorporating academic knowledge in mHealth platforms, funding will become more accessible, and the impact of these programs at a population level will increase, furthermore reinforcing expanded opportunities for investigation.

## Appendix

Multimedia Appendix 1 Video walkthrough of Sense Health platform.

Multimedia Appendix 2 Original grounded theory-structured interview guide.

Multimedia Appendix 3 Demographic characteristics of New York State Medicaid population in 2014 (N=4,534,400).

Multimedia Appendix 4 Complete codebook developed through grounded-theory methodology for analysis of care manager interviews.

## Abbreviations

mHealth: mobile health
MI: motivational interviewing
SCT: social cognitive theory
TTM: transtheoretical model
