# Supplementary material for: Investigating the Perceptions of Care Coordinators on Using Behavior Theory-Based Mobile Health Technology With Medicaid Populations: A Grounded Theory Study
Source: JMIR Mhealth Uhealth. 2017 Mar 21;5(3):e36. doi: 10.2196/mhealth.5892 (PMC5380813; doi:10.2196/mhealth.5892)
Supplement: Multimedia Appendix 2 [file mhealth_v5i3e36_app2.pdf]

Care Managers and Sense Health Interview Guide

1. Theme of Research
The purpose of this study is to explore how the integration of health behaviour theory into Sense Health, as a mobile health (mHealth) platform, influences engagement in Medicaid patients. These interviews are meant to explore the perception of patient navigators/care coordinators on the integration of Sense Health technology in their work with Medicaid populations, which will help provide us with context for our other data. At this stage in the research, their work is defined as helping their clients access, i.e. navigate the health care system in terms of their health issues.

Sub-questions:
--> How is technology perceived in relation to their own role in the patient-healthcare provider relationship?
--> How do care navigators perceive their patients' reaction to the technology?
--> Are there consistent pros and cons that come up that can be integrated into the development of mobile communication systems used in the healthcare industry?

2. Informed Consent

3. Interview
Background Information:
Can you tell me a little a bit about how you got to this job?
- What were you doing before?
- What did you study?
- What made you interested?

Role of patient navigator/care coordinator:
1. How would you describe your job?
    a. What are your roles and/or responsibilities?
2. How many clients do you manage?
3. What interferes with your ability to assist clients in obtaining/maintaining their care plan goals?
4. What helps facilitate the care management you provide?

Client Interactions
5. How would you describe your target patient population?
6. What are your clients' greatest needs when it comes to navigating the healthcare systems?
    a. or in another words, when it comes to making sure they know how to use the healthcare system to address their needs?
7. What are their greatest strengths (what do they find easiest to do) when it comes to using the healthcare system?
8. How responsive are patients to contact requests?
    a. During interactions?
9. What makes it difficult for clients to follow their care plan goals?

Navigator perception of Sense Health technology

10. How does the Sense Health platform fit in with the job responsibilities you mentioned before?
    a. Do you think there is a role for technology? Why/why not?
    b. Pros/cons?
    c. Would it be different with a non-Medicaid population?
11. What kinds of text messages do you send to clients?
    a. What do you like/find helpful about text messaging?
    b. What don't you like or find helpful about texting?
12. How does the SenseHealth program affect client engagement with their care plan goals?
13. Do you think the SenseHealth program improves the client's understanding of their condition(s) and how to better manage their health?
14. What would your job look like if you didn't use Sense Health?
15. Can you describe any ways you think technology can be used in a way it currently is NOT, to help your patients in your role as a patient navigator?
16. Can you describe the influence of using mobile tech on your relationship with patients?
    a. Would there be differences with a non-Medicaid population?
    b. *If you have not always used Sense Health while at this job, how have your interactions with your clients changed?*

Views on patients' perception of technology

17. How do clients react to using mobile technology?
    a. *(Are there any differences if it's a new client to whom you're introducing technology or if it was an old client who had to be newly introduced to the technology?)*
    b. Are there any differences based on the age, gender, or race of clients?
    c. Would there be differences with a non-Medicaid population?
18. Have your patients mentioned any ways technology can be used to help them navigate the health care system that aren't already in place?
    a. Either existing technology, or technology that doesn’t exist

19. Is there anything you would like to add that I didn't ask you about?
