# Supplementary material for: Investigating the Perceptions of Care Coordinators on Using Behavior Theory-Based Mobile Health Technology With Medicaid Populations: A Grounded Theory Study
Source: JMIR Mhealth Uhealth. 2017 Mar 21;5(3):e36. doi: 10.2196/mhealth.5892 (PMC5380813; doi:10.2196/mhealth.5892)
Supplement: Multimedia Appendix 3 [file mhealth_v5i3e36_app3.pdf]

Multimedia Appendix 1: Demographic characteristics of New York State Medicaid population in 2014 (N = 4,534,400)

Characteristics		%
Race/ethnicity	White	39
	Black	19
	Hispanic	30
	Other	23
Gender	Female	56
	Male	44
Age	0-18	43
	19-64	57
Family Work Status	At least 1 full-time worker	65
	Part-time workers	12
	Non-workers	23
Federal Poverty Level	Under 100%	33
	100-199%	32
	200-399%	21
	400%+	14
