# Supplementary material for: Investigating the Perceptions of Care Coordinators on Using Behavior Theory-Based Mobile Health Technology With Medicaid Populations: A Grounded Theory Study
Source: JMIR Mhealth Uhealth. 2017 Mar 21;5(3):e36. doi: 10.2196/mhealth.5892 (PMC5380813; doi:10.2196/mhealth.5892)
Supplement: Multimedia Appendix 4 [file mhealth_v5i3e36_app4.pdf]

Appendix 1: Codebook for Qualitative Interviews

Variable Name	Second-level code	Third-level code	Definition	Inclusion criteria	Exclusion criteria	Example
**Background**	Education		Interviewee's own background	Mentions own education background	Mentions others' background, present job, or future plans	"I started working here in December of last year, so I haven't been here a year yet. Previous similar work experience was with an undocumented population, pregnant women, and connecting them to services like Medicaid, and insurance, and teaching workshops for a community-based health center."
	Work experience					
**Job description**	Role		Description of job responsibilities	Definition of role at work, including different responsibilities, likes, dislikes, and barriers and facilitators of described role	Factors unrelated to job that influence job performance, as per role defined	"I have 60 clients on my caseload, and about half are HIV+, and about half – you have to have 2 chronic conditions to qualify for the program, so that's the sum of this organization. Oh, and harm reduction too I guess. We also focus on substance abuse and a harm reduction approach."
	Favourite part					
	Least favourite part					
	Barrier					
	Facilitator					
**Client description**	Demographics		Description of interviewee's clients	Demographic information, and needs and strengths of clients in care coordinator setting	Assumptions, description of how care manager wishes clients would act	"we do have a lot of clients with substance abuse issues"
	Needs					
	Strengths					
**Perception of technology use**	Of self	Positive	Perspectives on care managers and patients using technology	How care managers perceive the use of technology in their role, and how their perceive their patients use technology in	Perception of using phone calls or letters/other means to communicate; discussion of the type/content of technology use;	"they can text me about something, and I can help bring that up to their provider or remind them to"
	Of patient	Negative				

				their interactions; beliefs, attitudes	discussion of skills	
**Technology/ texting integration**	Self	Barrier	Integrating texting or other types of technology into the care manager/client relationship	Care managers talking about themselves, or their clients, integrating technology into their regular routines, and the barriers/facilitators, pros/cons, and areas of improvement for doing so	Reference of technology not relative to regular routine that previously didn’t use technology	“clients nowadays do text, and they have smartphones, so they can”
	Patient	Facilitator				
		Advantage				
		Disadvantage				
		Area for improvement				
**Medicaid**	Relevance		Mention of Medicaid	Medicaid is relevant to what is being mentioned	Medicaid is not mentioned by name	“A lot have what we call the Obama-phone, it’s a phone that I guess if you have Medicaid and you’re eligible for it, it’s a phone that you get for free with x amount of minutes.”
**Access to services**	Health-related	Need help	Discussion of clients’ access to services	Services that may or may not be accessible to clients	Opinion of services, or people who work at these services	“He was in the ER, he went to his primary care physician and he was sent to the ER just to have an ultrasound to follow up on a fall he had. And he was waiting, just like a regular ER patient. Like why wouldn’t he just be sent directly to radiology? It’s just sometimes some of the hospitals and clinics, they don’t have the right flow down, they’re not patient-centered.”
	Legal	Received/not				
	Other					
**Healthcare system**	Aspects with which clients have difficulty	Strategies to cope with difficulties	Reference to healthcare system	All components of healthcare system, parts that impede/ promote	Reference to other political systems, resources outside of	“I go to many hospitals all over NYC and so if your client is in good hands with a hospital that’s run properly and good administration, then you can feel more
	Factors that impede patient					

	engagement			engagement, how to make use of healthcare system less difficult	system that help with health	comfortable.”
	Factors that promote patient engagement					
	Culture					
**Care manager-client relationship**	Quality	Barriers	Reference to relationship between care manager and their clients	Quality of relationship, stories related to relationship, factors that can improve or worsen the relationship	Relationship between anyone else	“Ya they’ll want to leave, waiting for hours, so you’re kinda there to occupy their time.”
		Facilitators				
**Communication**	Method	Initiated by provider	Discussion of communicating with clients	Mention of how providers and patients communicate, the quality of the communication, if the efforts are reciprocated, barriers or facilitators of communication	Communication at appointments, or communication between clients and other people or care managers and others	“they can text me about something, and I can help bring that up to their provider or remind them to”
	Quality	Initiated by patient				
	Quantity	Balance				
	Difficulty/Ease					
	Reciprocity					
	Satisfaction with client response					
**Care plan goals**	Adherence		Reference care plan goals	Care plan goals are mentioned; adherence to them, barriers to adherence	Other life goals that are not set with care manager	“I guess the largest interference is maybe them not wanting to themselves.”
	Difficulties					
**Engagement**	With healthcare system	Barriers	Discussion of patients’ ability to interact, participate, and make proper use of defined subject matter	Engagement with healthcare system, care manager, technology	Other client characteristics, description of healthcare system itself, opinions on technology use	“It all depends, we offer a lot of support groups, we offer a cooking class, a sketch class, there’s other counselors here, like a harm reduction counselor, so some clients are engaged. It’s best when you can [get] clients engaged in other things the agency has to offer – that would be wonderful, because then they’re
	With care manager	Facilitators				
	Relationship to technology (in general)					

						more supportive, more supported."
**Sense Health**	Pros		Mention Sense Health platform	Sense Health platform tools, benefits and disadvantages of platform, what's missing, influence on clients understanding health condition or health information, its usefulness, introducing the technology to clients	Other technology, interactions with care manager or healthcare systems that do not involve Sense Health	"and now with Sense Health, prior to Sense Health it was difficult because a lot of what we were doing before was either using our own cell phones to get into contact with clients or when we were in the field"
***most used**	Cons					
	Relationship to engagement	Yes/No				
	Relationship to understanding of health condition/information					
	Utility	Room for improvement				
	Ease of use/ understanding					
	Transition					
**Trust**	Care manager-client		Reference to trust as a notable characteristic	Trust of client, client's trust of care manager, trust of health or justice system	Other feelings of care manager or client that aren't trust	"Ya, or some clients, they won't communicate another symptom they're having that they felt comfortable telling me."
	Healthcare provider-patient					
	Systemic (health/legal)					
**Difficulties with heavy caseload**			Reference to quantity of clients	Care managers experiencing difficulty due to the number of clients on caseload	Discussion of client characteristics	"It's hard when we're stretched thin, when we're maybe expected to go to the HRA, for something like a food stamp issue, or a housing issue, to go … apartment-viewing and stuff, when I have another client with an appointment that I want to make sure they understand about their diabetes."
**Separation of personal/ professional**	Ethics		Reference to distinction between personal life and professional life	Collision of personal and professional lives, ability to separate personal and	Challenges or stories related to either personal or professional life but not both	"sometimes the client saves that number and calls you or texts you at midnight on your personal phone so that was very common"

				professional lives		
**Appointment attendance**			Reference to clients going to appointments	Ability for clients to attend appointments, health or otherwise	Adherence to other parts of care plan goals, client characteristics	“Definitely finding out about their appointments, we do keep track whether they went to their appointments last month, if they have any coming up”
**Health as patient concern**			Discuss health as a concern	Health is mentioned, regardless of level of importance attributed	Other factors mentioned as a concern to patients	“Some people have really serious health issues.”
**Financial concerns**			Mention of client finances	Discuss how client finances impact decisions	Other concerns that impact client decisions	“But a lot of our clients, their number’s out of service, just because it’s hard to keep up with paying the bill, and they’ll get a different phone line, sometimes I have clients that every month it’s a different number.’
**Text length**			Reference to how long a text message can be	Discussion of length of text	Discussion of content of text	“I want my texts to be longer! (laughter) It’s very short, it’s like a tweet, so I just have to send it in chunks and I don’t know what it looks like on their end, when I something in chunks, you know it’ll cut off a phone number or something of someone they need to call.”
**Consent**			Mention of consent process	Talk about the consent process, whether how care manager perceives it or how clients perceive it	Other parts of using Sense Health technology or technology generally, or other aspects of client-care manager relationship	“We’ve all concluded that our biggest barrier to consenting clients is doing it over the phone.”
**Literacy levels**			Mention of	Literacy levels	Client	“And I do have a lot of clients with

			clients' literacy levels	and/or education status of clients	background not related to education and/or reading abilities and comprehension	low literacy levels, that's actually why a lot of my clients need advocacy, because they don't read or write."
**Seniors texting**			Discussion of elderly people using text messages	Mention of how people over 65+, or perceived as old, text and/or use technology	People of other age groups using technology	"Ya, definitely age plays a big factor."
**Provider-care coordinator dynamic**			Reference to interaction between care managers and healthcare providers	Interactions between healthcare providers and care coordinator	Interactions between care coordinator and other professionals, relationship between client and healthcare provider	"I have to advocate a lot to have my client see the same provider every time, to be assigned one primary care physician, and not see someone different every time, a different student, that's big here."
**Client culture/ Language**			Reference to client's cultural background and the language they speak	Mention of client's culture and/or language (likely other than English)	Other aspects of client demographic information	"I have a few like that and I have clients that are Spanish-speaking only and they don't always text and they don't always, they're illiterate."
**Willingness**			Open to changing health behavior	Discusses intent to change behavior, to make progress in terms of self-management or engagement, or referral to past occurrence of the same	Willingness to change other, non-health, behaviors	"It all comes down to them, personally, again, are they willing to do something? Are they willing to make that change? The ones who will, you'll see that change in them. Then the others, it's been the same story for a year or two."
**Accountability**			Responsible to one's actions	Reference to clients' accountability to	Accountability of technology	"It does give them a little bit more of, like, 'Okay, I have to do this because she's going to know if I

				their care manager or to their health, or to adherence, or care managers' accountability to their clients		don't.'"
**Support**			Sense Health's influence on being able to support clients, or clients being able to feel supported	Relates to support via technology	Care manager-client relationship independent of technology	"They know that there is that trust because already they've given me, they're allowing me to text them. It builds more trust and it builds more ... Like, they'll know that they can rely on me and so that's a part of it that's also good. They also, sometimes they like, "Oh, hey, thank you so much." It's very receptive."
**Motivation**			Desire to engage in health	Enthusiasm and incentives for changing health behaviors	Enthusiasm for changing non-health behaviors	"…the ones who I have their medication reminders for, like seeing that change in themselves, they're happy about. I guess it kind of helps them just be happier about what's going on in their life."
